# Cost implications of the physiotherapy management of complex tibial fractures treated with circular frames

**DOI:** 10.1007/s11751-013-0173-8

**Published:** 2013-08-13

**Authors:** E. Barron, R. Rambani, H. Bailey, H. K. Sharma

**Affiliations:** 1 Hull and East Yorkshire Hospital NHS Trust, Hull, UK; 2 Leeds Teaching Hospital NHS Trust, Sheffield, UK

**Keywords:** Cost analysis, Physiotherapy cost, Complex tibial fractures

## Abstract

Seventy-three consecutive patients with complex tibial fractures treated with an Ilizarov frame or Taylor Spatial Frame received physiotherapy between April 2008 and April 2010. Data were collected prospectively, and physiotherapy input was recorded (in minutes) for the patients identified. This included treatment received as an inpatient as well as an outpatient. The data were categorized for proximal, middle and distal third tibial fractures for analysis. The average cost of physiotherapy for an inpatient with an Ilizarov frame is £121.82 per case, whereas that for an outpatient receiving treatment for trauma was calculated as £404.60. The combined average cost of physiotherapy to support treatment of a complex tibial fracture with a fine wire fixator is £546.27. Treatment involving circular frames is complex and expensive, and the high physiotherapy cost is not reflected in Healthcare Resource Group codes. This cost calculation will help service units, and NHS Trusts develop realistic costing plans to support treatment. Cost implications of the physiotherapy management of complex tibial fractures using the Ilizarov technique.

## Introduction

Complex tibial fractures are difficult; Ilizarov frames are often used as definitive fixation, and patients require multidisciplinary input for rehabilitation [1, 2]. The cost implications of managing such injuries are significant for the hospital and patient in view of the considerable investment in time and resources [3].

The Physiotherapist is an essential member of the multidisciplinary team for treating these complex injuries [4]. The intensity of physiotherapy treatment needed varies for different types of injuries and fractures. The availability of the optimum input might affect patient outcome including reduced morbidity and improved functional status [5]; e.g., the amount of physiotherapy differs in a simple distal radius fracture to trauma patients with multiple long bone fractures requiring circular frames [4]. It is uncertain if there are commensurate resources in health care funding to account for this variation, and if increased payment is made to the specialist physiotherapy units that help in rehabilitating such patients.

No previous studies have been published evaluating the cost of physiotherapy in treating this patient group. The aim of this study is to assess the cost of physiotherapy intervention to patients with a circular (Ilizarov or Taylor Spatial) frame.

## Materials and methods

One hundred and seven patients underwent surgery with circular frames (Ilizarov and Taylor Spatial) in a 2-year period from April 2008 to March 2010. At the cutoff point of data collection, 73 patients had completed treatment and were eligible. After physiotherapy as an inpatient, 29 patients went on to receive outpatient physiotherapy within the Trust. The remaining 44 tertiary referrals had surgery in our hospital but were referred back for outpatient physiotherapy at the referring hospital; this prevented data collection for those patients. All data were collected prospectively. It consisted of contact time with the physiotherapist over the identified period, both contact time and the seniority of the treating physiotherapy (NHS agenda for change pay rate bands). The data were collected for inpatient and outpatient physiotherapy sessions. The average cost of treatment was calculated for each patient based on an estimate for each band of staff treating and for that financial year. This was provided by the Finance department of our Trust.

## Results

The tibial fractures were categorized for tibial plateau, tibial shaft, distal third/pilon and segmental fractures (Table 1). Of the 29 patients who were referred to outpatient physiotherapy within the trust 19 patients (65.5 %) completed their course of treatment. The remaining 10 patients were discharged from the physiotherapy service following non-attendance and subsequent failure to contact the physiotherapy department for further treatment. Eleven patients (57.9 %) had an Ilizarov frame, whereas 8 (42.1 %) had a Taylor Spatial Frame. The average time to union overall was 5 months and 21 days. The average time to union by type of fracture is shown in Table 1. There were no nonunions. The pin site infection rate was 52 %.

**Table 1:** Classification of tibial fractures

Type of fracture	Number	Average time to union
Tibial plateau and proximal third	3	5 months
Tibial shaft	5	5 months 21 days
Distal third and pilon	10	6 months and 24 days
Segmental	1	5 months 15 days

### Physiotherapy treatment time

The mean inpatient treatment time from preoperative assessment through to discharge home for a patient was 7.45 h (Table 2). This equates to a cost of £121.82 per patient. The average outpatient time for all the trauma patients who completed their course of physiotherapy was 20.21 h (Table 3). This equates to a cost of £404.65. The average number of treatment sessions per patient for this group was 33. These cost calculations include physiotherapist pay only but does not take into account the cost for providing the infrastructure and equipment used.

**Table 2:** Mean treatment time for inpatient trauma patient and physiotherapist banding

Physiotherapist banding (hourly rates)	Mean treatment duration per patient (h)	Cost (£) per patient
3 (£10.82)	1.74	18.82
5 (£15.12)	2.24	33.87
6 (£18.91)	2.51	47.46
7 (£22.58)	0.96	21.67
Total	7.45	121.82

**Table 3:** Mean treatment time and cost per trauma patient and physiotherapist banding (including patients who completed their course of treatment *n* = 19)

Physiotherapist banding (hourly rates)	Mean treatment duration per patient (h)	Cost (£) per patient
3 (£10.82)	3.38	36.57
5 (£15.12)	2.26	34.17
6 (£18.91)	3.52	66.56
7 (£22.58)	11.84	267.35
Total	20.21	404.65

## Discussion

This study has confirmed the significant physiotherapy resources required to rehabilitate patients with an Ilizarov or Taylor Spatial Frame (TSF). The average number of outpatient treatment sessions for routine trauma (non-circular frame) patient is 6 which, in contrast, is 33 for patients with a circular frame.

The overall average cost (including inpatient and outpatient physiotherapy) of rehabilitating a patient with a circular frame is £526.47 for trauma. The brunt is for outpatient physiotherapy where the pay costs for a physiotherapist averages £404.65. This is in comparison with other tibial fractures treated by internal fixation which cost £60.29 (when treated by a Band 7 physiotherapist) for outpatient sessions.

Patil and Montgomery reported an average cost of £30,000 of treating tibial and femoral nonunions using Ilizarov method [6]. As there are no Healthcare Resource Group (HRG) codes for reconstructive procedures using a circular frame or similar device, hospital Trusts which provide such tertiary level services do not get commensurate reimbursement for either surgery, device or rehabilitation costs. There is a need to develop patient-level costing, and subsequently, HRG codes for such complex treatments using circular frames for, in the absence data, hospitals managing these injuries would bear a substantial financial loss.

## Conclusion

The overall average cost (including inpatient and outpatient physiotherapy) of rehabilitating a patient with a circular frame is £526.47 for trauma. This data form part of the total patient—level cost that will enable hospitals in the United Kingdom to negotiate with commissioners for the extra resources needed to enable continued delivery of specialist care for complex fractures.
